# Child- versus adult-perspective composite time trade-off valuations for the EQ-5D-Y-3L: evidence from the Hungarian valuation study

**DOI:** 10.1007/s10198-025-01857-5

**Published:** 2025-10-30

**Authors:** Stevanus Pangestu, Bram Roudijk, Fanni Rencz, Stefan A. Lipman

**Affiliations:** 1 Department of Health Policy, Corvinus University of Budapest, Budapest, Hungary; 2 EuroQol Research Foundation, Rotterdam, the Netherlands; 3 Erasmus School of Health Policy and Management, Erasmus University Rotterdam, Rotterdam, the Netherlands

**Keywords:** EQ-5D-Y-3L, Health state, Perspective, Composite time trade-off, Valuation, Youth, I10

## Abstract

**Background:**

The EQ-5D-Y-3L is a generic, preference-accompanied health measure intended for pediatric populations. EQ-5D-Y-3L health states are valued using the perspective of a hypothetical 10-year-old child (‘child perspective’) rather than adults valuing for themselves (‘adult perspective’). The perspective used has been shown to influence valuation outcomes, affecting comparability of health utilities. This study explored within-respondent differences in values between adult and child perspectives using data from Hungary.

**Methods:**

A secondary analysis was conducted using composite time trade-off (cTTO) data from the Hungarian EQ-5D-Y-3L valuation study. Two hundred adults valued 10 health states from the child perspective and four from the adult perspective. The cTTO values for the matched health states (valued from both perspectives) were compared, with differences analyzed using t-tests and random-intercept regression. Associations with respondent characteristics were also explored.

**Results:**

Differences in cTTO values were observed between perspectives, particularly for more severe health states. Compared to the adult perspective, the child perspective yielded significantly lower values for worse-than-dead observations, but higher values for better-than-dead observations. After adjusting for within-subject variation and respondent characteristics, perspective was not a significant predictor of cTTO values. Instead, differences were partly explained by education, region of residence, parental status, and the view that a child’s life is more valuable than an adult’s.

**Conclusions:**

This is the first study to explore perspective differences in EQ-5D-Y-3L health state valuation within respondents using nationally representative data from outside Western Europe. The findings highlight the importance of considering individual-level attributes in pediatric health valuation.

**Supplementary Information:**

The online version contains supplementary material available at 10.1007/s10198-025-01857-5.

## Introduction

The EQ-5D-Y-3L is a standardized, generic, preference-accompanied instrument for measuring health-related quality of life in children and adolescents aged 4 to 15 [1, 2]. It is an adaptation of the EQ-5D, designed to be comprehensible for younger populations. To develop national value sets for the EQ-5D-Y-3L, which are necessary for calculating quality-adjusted life-years (QALYs), the EQ-5D-Y-3L International Valuation Protocol recommends that members of the general population value hypothetical child-specific health states using both composite time trade-off (cTTO) and discrete choice experiment (DCE) methodologies [3]. A key difference from the valuation of the adult EQ-5D instrument is that for the EQ-5D-Y-3L, adults are interviewed to elicit preferences from the perspective of a hypothetical 10-year-old child (hereafter referred to as the child perspective), rather than from their own point of view (hereafter: the adult perspective). As acknowledged by the authors of the protocol [3], asking adults to value children’s health states (i.e., to express preferences about which health state would be better for a 10-year-old child) presents both methodological and normative challenges [4]. Key concerns include whether adults can accurately represent children’s experiences and the ethical importance of involving children themselves to respect their right to express their views [5, 6]. Nevertheless, the use of the child perspective by adults was introduced to reflect a taxpayer view and to address concerns regarding the complexity of the valuation tasks for children [3].

Previous research has illustrated how varying perspectives in cTTO can elicit different responses. Such differences may affect the comparability of health state valuations, potentially compromising standardized value sets and leading to biased QALY calculations [4]. Generally, health state values for children are higher than those for adults [3, 7, 8], with higher valuations linked to less variability [8]. Qualitative evidence suggests several reasons for the reluctance to trade-off life years in children: discomfort with imposing hypothetical impairments on children, the perception that adults cope better with difficulties, and uncertainty over who is best suited to make these valuations [9–11]. It has been suggested that adults may find it more challenging to value health states using a child’s perspective due to adults perceiving children’s health states as more similar to one another, or using simpler heuristics when considering children’s health-related quality of life [12].

Some studies have examined how the same individual (i.e., within respondents) values EQ-5D-Y-3L health states from different perspectives. One experiment with 200 Dutch university students showed that valuation outcomes can systematically vary depending on whether respondents valued health states from the perspective of an adult or a child, and whether they were deciding for themselves or for another person [8]. Another experiment involving 150 Dutch adults found no significant differences between valuing child health states using the adult and child perspectives [13], whereas two studies with UK adults identified significant differences between these perspectives [14, 15]. Such differences in utilities between both perspectives were even found to be more pronounced in lead-time TTO compared to cTTO [16].

Despite recent findings on within-respondent differences, further research is needed to better understand how perspective-driven differences influence utility values. Existing studies comparing cTTO perspectives have been conducted primarily in Western Europe [8, 14–18]. It is important to determine whether issues related to different perspectives extend across different countries and cultures, as health utilities may vary based on cultural values and geographical contexts [19–22]. In addition, previous research has shown that valuation outcomes can also be influenced by individual characteristics such as age, sex, and education [13, 23]. This study aims to contribute to the literature by providing insights from Hungary, a Central and Eastern European country where the EQ-5D and EQ-5D-Y-3L are preferred instruments for health technology assessment [24]. Our analysis is grounded in data collected using the EuroQol Valuation Technology (EQ-VT) as part of the EQ-5D-Y-3L valuation study in Hungary [25].

## Methods

As the study methodology, including preference elicitation methods, health state selection, and sampling, has been described in detail elsewhere [25, 26], this section provides only a brief overview of the original methods.

### EQ-5D-Y-3L

The EQ-5D-Y-3L consists of a descriptive system and a visual analogue scale (EQ VAS). The descriptive system assesses health-related quality of life across five dimensions: mobility (walking about), looking after myself (washing or dressing), usual activities (going to school, hobbies, sports, playing, doing things with family or friends), having pain or discomfort, and feeling worried, sad, or unhappy. Each dimension has three response levels: level 1 indicates ‘no problems’, ‘no pain or discomfort’, or ‘not worried, sad, or unhappy’; level 2 indicates ‘some problems’, ‘some pain or discomfort’, ‘a bit worried, sad, or unhappy’; and level 3 indicates ‘a lot of problems’, ‘a lot of pain or discomfort’, or ‘very worried, sad, or unhappy’. A respondent’s health state profile is represented as a five-digit string, where each digit corresponds to the severity level in a given dimension. For example, ‘12323’ indicates no problems with walking about, some problems with washing or dressing, a lot of problems doing usual activities, some pain or discomfort, and very worried, sad, or unhappy. Since each dimension has three levels, there are 243 (3^5^) unique health states. A level-sum-score (LSS) can be calculated by summing the five-digit health state, with possible scores ranging from 5 (full health: 11111) to 15 (worst health: 33333). Next, the EQ VAS captures self-rated health on 0–100 vertical scale, where 0 and 100 represent ‘the worst health you can imagine’ and ‘the best health you can imagine,’ respectively. Existing evidence on the measurement properties of the EQ-5D-Y-3L supports its applicability [27]. This study used the official Hungarian version of the EQ-5D-Y-3L.

### Data description

A secondary analysis was conducted using data collected from the Hungarian EQ-5D-Y-3L valuation study, which received ethical approval from the Research Ethics Committee of Corvinus University of Budapest (KRH/31/2021) [25]. The cTTO tasks were carried out using the EQ-VT (v2.1) software, which included both conventional 10-year TTO valuations better-than-dead states and a lead-time TTO variant for worse-than-dead states (i.e., 10 years in full health followed by 10 years in an EQ-5D-Y-3L state). The interviews were conducted by four graduate students who had prior experience with the Hungarian EQ-5D-3L and EQ-5D-5L parallel valuation study [28]. All interviewers received standardized training on valuation methods, the EQ-VT protocol, and quality control procedures. Each interviewer completed 50 interviews for the EQ-5D-Y-3L valuation study.

Each recruited respondent valued two example health states (e.g., being in a wheelchair), three practice EQ-5D-Y-3L health states (21112, 32323, and 13311), and 10 ‘real’ EQ-5D-Y-3L states from the perspective of a 10-year-old child (exact phrasing: ‘Considering your views for a 10-year-old child.’). Respondents valued the following 10 health states in random order: three mild states (11112, 11121, and 21111), two moderate states (22223 and 22232), four severe states (31133, 32223, 33233, and 33323), and the worst health state (33333). After completing the valuation tasks, respondents were presented with a ranked list of the 10 health states (‘feedback module’) based on their responses. Using this module, respondents had the option to flag any health state valuation that they felt did not reflect their preferences, even if the responses appeared consistent. As the final step of the interview, respondents valued another four EQ-5D-Y-3L health states, this time from their own (adult) perspective. These states were randomly selected from the same set of 10 health states previously valued from the child perspective. The ‘feedback module’ was not used for the adult-perspective valuations.

Overall, 200 Hungarian adults, representative of the general population in terms of age and sex, completed the cTTO tasks. In addition to the valuation exercise, respondents completed a questionnaire covering sociodemographic characteristics (e.g., education, civil status, number of children and their age, and residential area), health status (i.e., chronic conditions and self-rated general health), and self-complete versions of the EQ-5D-3L and EQ-5D-5L. They also rated their agreement with the statement: ‘A child’s life is worth more than an adult’s as they have more ahead of them,’ using a five-level Likert scale (‘strongly disagree’ to ‘strongly agree’).

### Statistical analysis

Each respondent valued 10 health states from the child perspective and four from the adult perspective, allowing for direct paired comparisons of the four matched health states valued from both perspectives. Prior to analysis, observations flagged in the EQ-VT ‘feedback module’ were excluded to ensure data quality. Distributions of cTTO values for the child and adult perspectives were first visualized separately using histograms. Clustering at the extreme values (−1.0 and 1.0) was compared between perspectives using McNemar’s test to confirm that the observed pattern was not due to random variation. The distribution of paired differences (i.e., child minus adult values) was examined using median and deciles, and the Wilcoxon signed-rank test was performed to assess whether the median differed significantly from zero. A Bland-Altman plot was generated to illustrate the difference between the two perspectives, displaying the difference (y-axis) against the mean value for each matched pair (x-axis) with points color-coded by LSS. Mean differences between perspectives were assessed using the Student’s t-test; mean cTTO values were initially compared by individual health state profiles and subsequently by severity categories (i.e., mild, moderate, and severe/worst) to improve statistical power. Subgroup analyses were additionally performed for better-than-dead and only worse-than-dead values, where the number of observations was sufficient. Classification was based on the child-perspective value; e.g., if a state was rated better-than-dead in the child perspective but worse-than-dead in the adult, it was classified as better-than-dead. cTTO values exactly equal to zero were classified as either better-than-dead or worse-than-dead, depending on the type of valuation task completed by respondents: conventional TTO for the former or lead-time TTO for the latter.

To explore predictors of differences in cTTO values between perspectives, four multivariate linear regression models were estimated. Random-intercept models were used to account for the repeated observations per respondent, and robust standard errors were applied to account for heteroskedasticity. In the first two models, cTTO values were regressed separately for the child and adult perspectives to examine perspective-specific associations, using all responses for each perspective (i.e., not limited to the matched observations). Predictors included the LSS of the valued health states (centered by subtracting six to simplify interpretation, given that its values ranged from 6 to 15), age, sex, education, region of residence, number of children, and the view that a child’s life is more valuable than an adult’s (recoded as a binary variable due to limited variability, with responses dichotomized into agreement versus disagreement or neutrality). Individual-level covariates were selected based on prior literature [19–22]. For the subsequent models, analyses were restricted to health states that had been valued under both perspectives. In the third model, cTTO values were pooled into a single regression and a dummy variable for perspective was included as an additional predictor (coded 1 for child perspective, 0 for adult). This specification allowed to test the overall effect of perspective, while controlling for the same set of respondent characteristics. In the fourth model, the same predictors (as in the first two models) were used to estimate differences in cTTO values, using the difference between the child and adult values for each matched health state as the dependent variable. The analyses were conducted using Stata/MP 18 (StataCorp LLC, 2023) and the Bland-Altman plot was generated using the ‘ggplot2’ package in RStudio 2024.12.1 + 563 (Posit Software, PBC). Statistical significance was set at *p* < 0.05.

## Results

Table 1 presents an overview of respondent characteristics, while comprehensive details have been previously published [25]. The study included 200 respondents (mean age 48.1 ± 18.6; 46.0% female). Most had at least a secondary education (71.5%), were married or partnered (64.5%), and had children (61%). Most respondents reported being in at least good health (80.5%). A total of 34% respondents agreed that a child’s life held greater value than an adult’s, while 28% were neutral and 38% disagreed.

**Table 1:** Respondent characteristics

Variables		Overall sample (*n* = 200)	
		*N* or Mean	% or SD
Age		48.1	18.6
Sex			
	Female	108	54.0%
	Male	92	46.0%
Highest education			
	Primary or less	57	28.5%
	Secondary	82	41.0%
	Tertiary	61	30.5%
Civil status			
	Married/partnered	129	64.5%
	Single	37	18.5%
	Divorced	8	4.0%
	Widowed	21	10.5%
	Others	5	2.5%
Children^a^			
	None	78	39.0%
	At least one < 18	45	22.5%
	Only child(ren) ≥ 18	77	38.5%
Net monthly household income (HUF)			
	50,001–500,000	48	24.0%
	500,001–800,000	38	19.0%
	800,000+	27	13.5%
	Didn’t know/refused to answer	87	43.5%
Residential area			
	Western Hungary	85	42.5%
	Central Hungary	49	24.5%
	Eastern Hungary	66	33.0%
Views child’s life as more valuable than adult’s			
	Strongly disagree	16	8.0%
	Disagree	60	30.0%
	Neutral	56	28.0%
	Agree	53	26.5%
	Strongly agree	15	7.5%
Self-rated general health			
	Excellent	35	17.5%
	Very good	69	34.5%
	Good	57	28.5%
	Poor	32	16.0%
	Fair	7	3.5%

^a^Included biological, adopted, and stepchildren

**Fig. 1:**
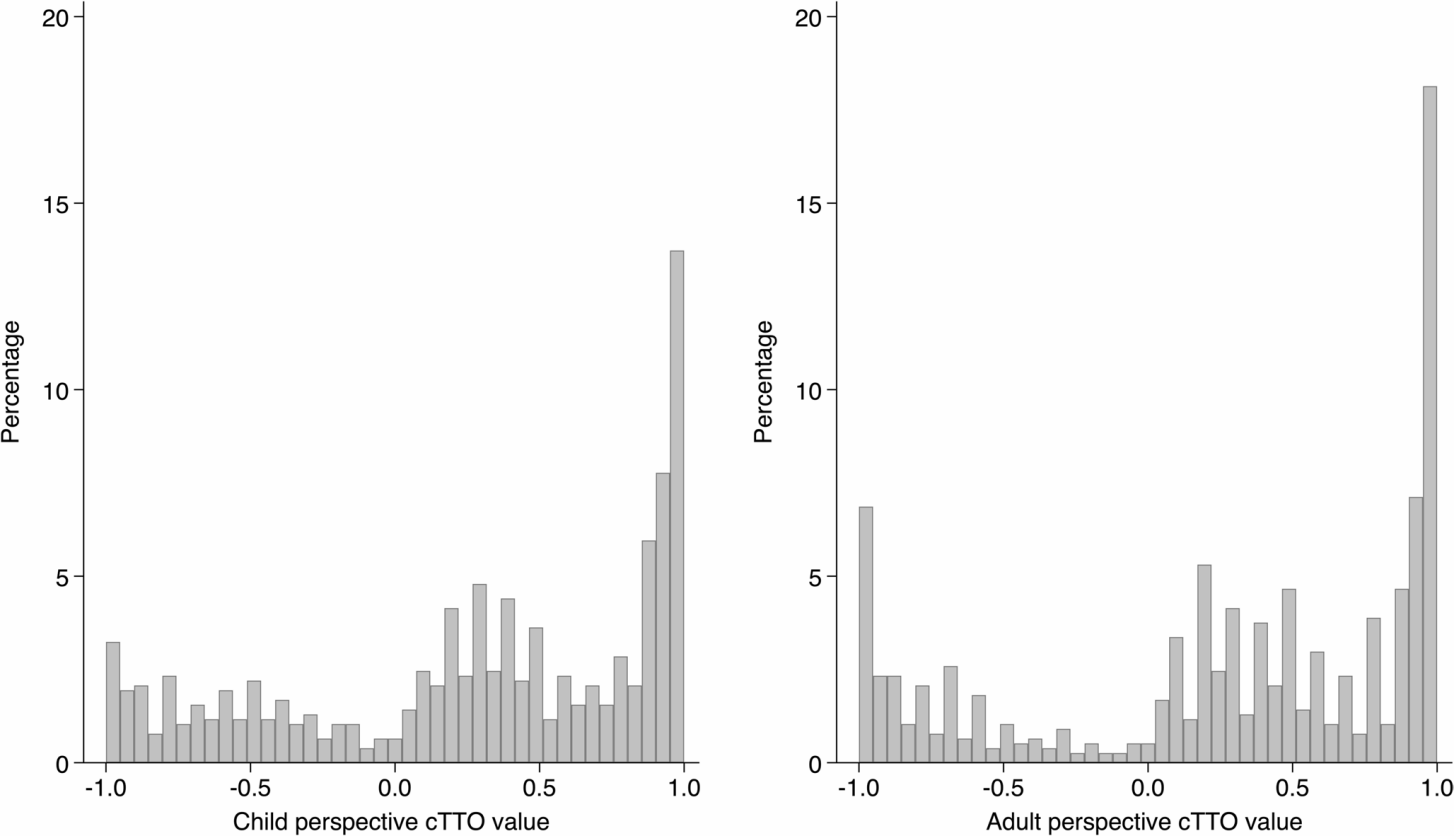
cTTO values for adult and child perspectives

**Fig. 2:**
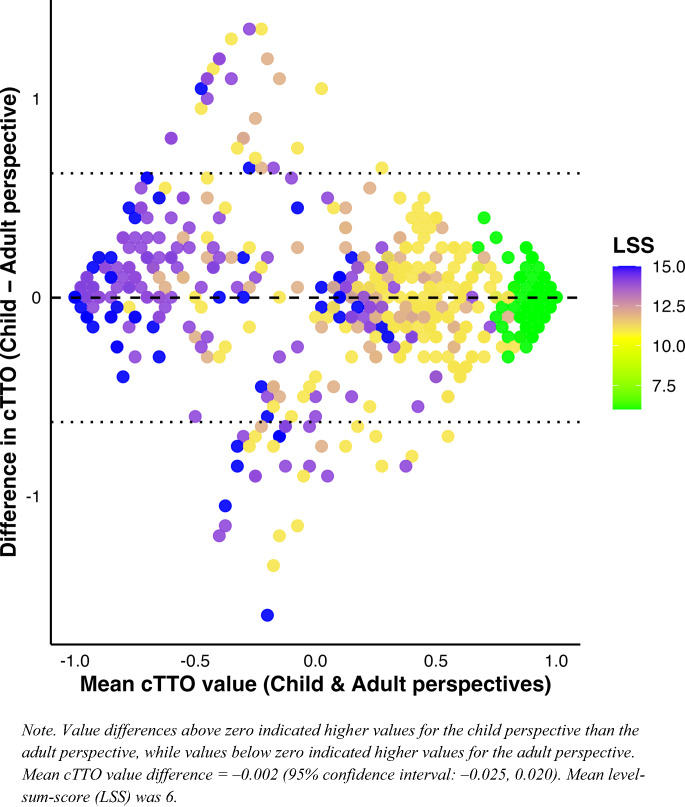
Bland-Altman plot assessing perspective differences

After excluding 68 flagged responses through the feedback module, 772 observations were included in the matched comparisons. Figure 1 shows the full cTTO distributions for both perspectives, displayed as percentages. The adult perspective showed more pronounced clustering at both 1.0 and − 1.0 (McNemar’s chi-square = 28.26, *p* < 0.001). The distribution of paired differences (child minus adult) was centered around zero, with 80% of observations falling between − 0.30 and 0.29. The Wilcoxon signed-rank test indicated no shift in the median of paired differences from zero (*p* = 0.458). Figure 2 illustrates that differences between perspectives were smaller for milder health states, but variability increased for more severe states, where child-perspective values tended to be higher than adult-perspective values. Further examination revealed that 33 observations were considered worse-than-dead in the adult perspective but better-than-dead in the child perspective, and conversely, 54 were considered better-than-dead in the adult perspective but worse-than-dead in the child perspective. These cases occurred primarily in severe states: 29 (87.9%) and 37 (68.5%), respectively.

The mean cTTO difference was close to zero (*p* = 0.839) (Table 2). For mild health states, mean cTTO values were slightly lower for the child perspective (mean difference=−0.01, *p* = 0.015). No significant differences were found for moderate (*p* = 0.128) or severe/worst (*p* = 0.348) health states. When considering only better-than-dead observations, the child perspective showed significantly higher values (mean difference = 0.03, *p* = 0.005), particularly for severe/worst states (mean difference = 0.11, *p* < 0.001). Conversely, in worse-than-dead observations, the child perspective had overall lower (or worse) values than the adult perspective (mean difference=−0.08, *p* = 0.003), including for severe/worst states (mean difference=−0.06, *p* = 0.042). Comparisons of cTTO values between perspectives by health state profile are presented in Table S1.

**Table 2:** Comparison of cTTO values between perspectives by health state severity

Health state		*n*	Child perspective^a, b^		Adult perspective^b^		Mean difference	*p*-value
			Mean	SD	Mean	SD		
All cTTO values								
	Mild	245	0.94	0.08	0.95	0.08	−0.01	0.015
	Moderate	145	0.39	0.34	0.43	0.36	−0.04	0.128
	Severe/worst	382	−0.19	0.54	−0.21	0.60	0.02	0.348
	Overall	772	0.28	0.64	0.28	0.68	0.00	0.839
***Better-than-dead states***								
	Mild	245	0.94	0.08	0.95	0.08	−0.01	0.015
	Moderate	129	0.48	0.21	0.47	0.30	0.01	0.798
	Severe/worst	177	0.32	0.20	0.21	0.44	0.11	< 0.001
	Overall	551	0.63	0.32	0.60	0.43	0.03	0.007
***Worse-than-dead states*** ^***c***^								
	Severe/worst	205	−0.63	0.28	−0.57	0.46	−0.06	0.042
	Overall	221	−0.61	0.28	−0.53	0.50	−0.08	0.003

*Abbrv.* *cTTO * composite time trade-off, *SD* standard deviation

^a^Excluding flagged responses.

^b^Only matched health states, i.e., valued from both perspectives. Better- or worse-than dead classification was based on the child-perspective value.

^c^Moderate health states with negative cTTO values (n = 16) were excluded due to insufficient number of observations.

*Notes.*1. The severity of valued health states were mild (11112, 11121, and 21111), moderate (22223 and 22232), severe (31133, 32223, 33233, and 33323) and worst (33333).

Severe and worst health states were combined in theanalysis. There were 87 observations for the worst health state, with 25 rated as better-than-dead and 62 as worse-than-dead. In the worse-than-dead category, the mean difference forsevere states alone was not statistically significant (mean difference= -0.05, *p*=0.170), suggestingthat the observed effect was primarily driven by the worse state. For both the full set of observations and the better-than-dead category, there results were consistent regardless of whether the worst state was included among the severe states.

Table 3 presents regression results on factors associated with cTTO values. In Models 1 and 2, most respondent characteristics were not statistically significant. In the adult-perspective model (Model 2), having at least one child (aged < 18) was associated with higher cTTO values (beta = 0.137, *p* < 0.05). Model 3 showed no significant effect of perspective on cTTO values (beta=−0.002, *p* = 0.871). Across all three models, the LSS of the valued health states was consistently a significant predictor (*p* < 0.001). Model 4, which analyzed paired differences between perspectives, found that greater value differences (i.e., higher values from the child relative to the adult perspective) were associated with respondents who agreed that a child’s life was more valuable than an adult’s (*p* < 0.05). Conversely, lower value differences (i.e., higher values from the adult perspective) were associated with possessing secondary education, residence in Central Hungary, and having only adult children (all *p* < 0.05), with the latter showing the largest effect (beta=−0.116). Sensitivity analyses including the flagged states in the feedback module showed no difference in the regression results.

**Table 3:** Regression coefficients and robust standard errors

Variable	Outcome: cTTO values			Outcome: cTTO value differences (child minus adult values)
	Child perspective	Adult perspective	Both perspectives^a^	Both perspectives^a^
	Model 1	Model 2	Model 3	Model 4
Constant	0.906 (0.073)^***^	0.783 (0.092)^***^	0.832 (0.083)^***^	0.107 (0.053)^*^
LSS of valued health state	−0.152 (0.003)^***^	−0.157 (0.005)^***^	−0.156 (0.004)^***^	0.003 (0.003)
Female	0.017 (0.038)	−0.049 (0.048)	−0.007 (0.042)	0.054 (0.029)
Age	0.000 (0.001)	0.001 (0.001)	0.001 (0.001)	0.000 (0.001)
Education (ref: primary)				
Secondary	0.038 (0.042)	0.116 (0.054)	0.071 (0.047)	−0.092 (0.032)^**^
Tertiary	0.029 (0.056)	0.073 (0.073)	0.056 (0.062)	−0.037 (0.046)
Country region (ref: Western Hungary)				
Central Hungary	0.007 (0.054)	0.105 (0.072)	0.035 (0.060)	−0.108 (0.048)^*^
Eastern Hungary	0.019 (0.039)	0.044 (0.052)	0.031 (0.045)	−0.017 (0.031)
Children (ref: no children)				
At least one child < 18	0.074 (0.045)	0.137 (0.062)^*^	0.093 (0.052)	−0.075 (0.043)
Only child(ren) > = 18	0.018 (0.049)	0.129 (0.071)	0.064 (0.059)	−0.116 (0.041)^**^
Views child’s life as more valuable than adult’s^b^	−0.016 (0.039)	−0.084 (0.048)	−0.048 (0.042)	0.067 (0.028)^*^
Child perspective	-	-	−0.002 (0.014)	-
n	1932	800	1544	772
R-squared	64.13%	60.28%	63.46%	5.12%

*, **, and *** indicate significance at *p* < 0.05, *p* < 0.01, and *p* < 0.001, respectively.

*Abbrv.* *cTTO* composite time trade-off, *LSS* level sum score, *ref* reference category

Note. Flagged responses were excluded from the analyses.

^a^Only matched health states, i.e., valued from both perspectives.

^b^Coded as 1 if respondents agreed with the statement, 0 if neutral or disagreed.

## Discussion

This study is the first to explore within-respondent differences in valuing EQ-5D-Y-3L health states from both adult and child perspectives in a national sample outside Western Europe. When comparing valuations of the same health states within respondents, we observed varied evidence regarding the influence of perspective. The child perspective yielded higher values for better-than-dead observations, a pattern consistent with findings from earlier studies [10, 15, 17, 18]. However, for worse-than-dead observations, the child perspective yielded lower values than the adult perspective. Across both better- and worse-than-dead observations, notable differences were observed across mild, moderate, and severe health states, with the gap widening as severity increased; a pattern also seen in previous research for the most severe states [8, 23]. For milder states, valuations tended to converge, perhaps because both children and adults are perceived as similarly able to manage minor health issues. Smaller differences in these cases may also reflect statistical constraints, as large variations are less likely when health states are closer to full health. As hypothetical health problems worsened, the emotional and cognitive burden of the valuation task likely increased. However, the worst health state might not have been the most cognitively demanding; moderate and severe states may have posed greater challenges, requiring more deliberation and nuanced trade-offs. This may help explain the contrasting pattern seen in severe states, where child-perspective values were higher when states were considered better-than-dead but lower when considered worse-than-dead. One possible explanation is the stronger emotional response evoked when imagining a child in extreme suffering. In addition, adults may project stronger time preferences onto children, potentially contributing to opposite valuation patterns in better-than-dead versus worse-than-dead scenarios [16, 29]. Respondents may have found it particularly distressing to consider a child experiencing severe illness, pain or disability, possibly due to the children’s greater perceived vulnerability compared to adults [9, 10], leading to more negative valuations in worse-than-dead cases. Conversely, when states were judged better-than-dead, the child perspective may have prompted greater optimism or a reluctance to trade life years, resulting in higher valuations. Alternatively, this pattern may reflect methodological effects; the use of lead-time TTO for worse-than-dead states could have introduced additional complexity, potentially influencing how respondents arrived at their decisions.

Interestingly, perspective was not significantly associated with cTTO values after controlling for within-subject variation in the regression analysis. This suggests that the observed differences may be attributable to individual-level attributes rather than perspective alone. Higher values from the child’s perspective were associated with those who held the view that a child’s life is more valuable than an adult’s. This view may reflect a normative or ethical stance that makes respondents more reluctant to trade off life years during valuation tasks. Meanwhile, respondents with secondary education, those living in Central Hungary, and with only adult children were more likely to assign higher values from the adult perspective. Residents of Central Hungary may exhibit more individualistic views, potentially influenced by higher income, different political views, greater healthcare access and urban social norms [30–32]. Likewise, parents of adult children may shift their preferences toward adult health issues [33], including concerns related to their own and that of their children. Our finding regarding education contrasts with a Dutch general population study, which found that higher educational attainment was associated with a greater prioritization of caring for children [13]. These differing public attitudes may reflect underlying cultural values and differences between national healthcare systems.

A recent Delphi study, which gathered input from experts across 18 countries, highlighted that there is still no clear consensus on the perspective to use when adults are asked to value child health states [34]. With the introduction of EQ-5D-Y-5L [2] and expected rollout of future valuation studies, the question of which perspective to adopt becomes increasingly relevant. Our findings confirm that the perspective used in valuation tasks significantly influences utility values. Such differences can affect the precision and comparability of health state valuations, introducing systematic variations that may impact health economic analyses and reimbursement decisions. However, these differences may not be uniform and vary across populations. To some extent, these variations in valuation outcomes appear to be shaped by cultural and sociodemographic factors. One possible direction for future valuation protocols is to offer a suite of standardized approaches that can be selected based on the health system context. Instead of applying a single fixed method, such a suite would allow decision-makers to choose the approach that best reflects local priorities and value considerations. Further methodological research is needed to determine the most robust approach, alongside stakeholder engagement to establish normatively acceptable valuation practices.

This study has several limitations. As a secondary study which relied on an existing dataset, some analyses may have been underpowered. All respondents valued health states from the child perspective before the adult perspective, introducing a potential order effect that may have influenced the results. This sequencing could have also led to learning effects, as respondents were already familiar with the valuation tasks by the time they reached the adult-perspective exercises [35]. Respondent fatigue may have emerged by this stage as well, potentially affecting the quality of responses. The limited overlap in health states valued from both perspectives may have also reduced variability in responses and constrained the scope of our comparisons. More broadly, the relatively small set of 10 states included in many EQ-5D-Y-3L valuation studies may have limited overall variation across respondents. This might have been different if a larger set of states had been presented across respondents, for example by using multiple blocks. Lastly, data collection took place during the COVID-19 pandemic, which may have influenced respondents’ health preferences. Despite these limitations, this study provides valuable insights into factors associated with cTTO value differences, using EQ-VT (v2.1) data that allowed for direct within-respondent comparisons.

## Conclusions

This study identified within-respondent differences in the valuation of child health states depending on the perspective used. However, after adjusting for within-respondent variation, perspective was not a systematic predictor of health state values. Instead, differences were more closely associated with individual-level attributes. These findings highlight the need for careful consideration in future valuation studies, as both the choice of perspective and cultural context can influence utility values and, subsequently, health economic evaluations.

## Supplementary Information

Below is the link to the electronic supplementary material.

Supplementary file1 (DOCX 8.77 MB)

## Data Availability

The data used in this study are available from FR upon reasonable request.
