# Supplementary material for: Child- versus adult-perspective composite time trade-off valuations for the EQ-5D-Y-3L: evidence from the Hungarian valuation study
Source: Eur J Health Econ. 2025 Oct 30;27(3):769–78. doi: 10.1007/s10198-025-01857-5 (PMC13190758; doi:10.1007/s10198-025-01857-5)
Supplement: Supplementary file 1 [file 10198_2025_1857_MOESM1_ESM.docx]

Electronic supplementary material

# Table S1. Comparison of cTTO values between perspectives by health state profile

| Health state | n | Child perspectivea,b | Adult perspectiveb | Mean difference | p-value |
| --- | --- | --- | --- | --- | --- |
| Mean | SD | Mean | SD |
| 11112 | 85 | 0.96 | 0.06 | 0.97 | 0.05 | -0.01 | 0.012 |
| 11121 | 78 | 0.91 | 0.09 | 0.94 | 0.09 | -0.03 | 0.034 |
| 21111 | 82 | 0.94 | 0.08 | 0.94 | 0.09 | 0.00 | 0.954 |
| 22223 | 73 | 0.49 | 0.30 | 0.48 | 0.26 | 0.01 | 0.756 |
| 22232 | 72 | 0.29 | 0.35 | 0.36 | 0.42 | -0.07 | 0.098 |
| 31133 | 72 | 0.08 | 0.46 | 0.07 | 0.55 | 0.00 | 0.959 |
| 32223 | 73 | 0.19 | 0.40 | 0.14 | 0.46 | 0.05 | 0.260 |
| 33233 | 74 | -0.38 | 0.49 | -0.40 | 0.59 | 0.02 | 0.796 |
| 33323 | 76 | -0.24 | 0.46 | -0.32 | 0.56 | 0.08 | 0.095 |
| 33333 | 87 | -0.52 | 0.50 | -0.48 | 0.56 | -0.04 | 0.307 |
| Overall | 772 | 0.28 | 0.64 | 0.28 | 0.68 | 0.00 | 0.839 |
| Abbrv. SD= standard deviation aExcluding flagged responses bOnly matched health states, i.e., valued from both perspectives. |
